# Polycystic ovarian syndrome elevates the distress of sexual pain in Iranian women with infertility

**DOI:** 10.1186/s12905-024-03181-1

**Published:** 2024-06-22

**Authors:** Bita Tahmasbi, Reza Eshraghi, Mohammadali Amini-Tehrani, Hadi Zamanian, Ashkan Ilami

**Affiliations:** 1 Shahid Akbarabadi Clinical Research Development Unit (ShACRDU), Iran University of Medical Sciences (IUMS), Tehran, Iran; 2 School of Medicine, Kashan University of Medical Sciences, Qotbe Ravandi St, Kashan, 8715981151 Iran; 3 Health Psychology and Behavior Medicine Research Group, Students’ Scientific Research Center (SSRC), Exceptional Talents Development Center (ETDC), Tehran University of Medical Sciences (TUMS), Tehran, Iran; 4 School of Psychological Science, University of Western Australia (UWA), Perth, Australia; 5 School of Public Health, Qom University of Medical Sciences, Lavasani St, Qom, 3713649373 Iran; 6 School of Psychology, Charles Sturt University, Victoria, Australia; 7 Kish International Campus, Tehran University of Medical Sciences, Tehran, Iran

**Keywords:** Infertility, Sexual dysfunction, Polycystic ovarian syndrome, Depression, Moderation

## Abstract

**Background:**

Sexual dysfunction may lead to sexual distress in women with infertility, while polycystic ovarian syndrome (PCOS) may escalate this distress. This study aimed to investigate the role of PCOS in the relationship between sexual dysfunction and sexual distress in Iranian women with infertility.

**Methods:**

The Female Sexual Function Index (FSFI), Female Sexual Distress Scale-Revised (FSDS-R), and Depression and Anxiety modules of the DASS-21 were cross-sectionally investigated in 190 women with infertility (103 women with PCOS and 87 women without PCOS).

**Results:**

There were negative correlations between sexual function domains and sexual distress (*P* < .001) in the total sample. Moderation analysis revealed that higher levels of impaired desire, arousal, and pain elevated sexual distress in the PCOS group. After adjusting for depression and anxiety, only the association between sexual pain and sexual distress was moderated by PCOS condition (*P* = .008).

**Conclusions:**

The findings suggest that impaired sexual function is associated with increased levels of sexual distress in infertile female patients. Importantly, comorbid PCOS renders patients susceptible to sexual distress where sexual pain is increased. Further research may shed light on the physiological, psychological, and relational aspects of sexual pain and associated distress in infertile female patients with comorbid PCOS.

## Introduction

Infertility is defined as the inability to achieve a clinically confirmed pregnancy after 12 months (age < 35 years) or six months (age 35 years and older) of regular unprotected penile-vaginal intercourse [1, 2]. While it is estimated to affect one-seventh of couples in Western countries, the prevalence of primary infertility in Iran, based on the World Health Organization’s clinical definition, is reported to be as high as 20.2% (i.e., one-fifth) [1, 3]. Women with infertility may suffer from various psychosocial problems, with sexual dysfunction and sexual distress among the primary distressing issues [4–6]. The prevalence of sexual dysfunction in women with infertility is high [7]. The current study sought to examine the worsening effect of polycystic ovary syndrome (PCOS) on the relationship between sexual dysfunction and sexual distress.

Polycystic ovary syndrome (PCOS) is one of the most prevalent endocrine problems and may cause ovulation failure, in which the ovaries and adrenal glands may produce excess androgens, resulting in an increased risk of infertility [8]. Based on different diagnostic criteria, the National Institutes of Health (NIH) or Rotterdam criteria, the prevalence of PCOS across Iran has been shown to be 3–7% [9]. PCOS has a complex relationship with infertility [10] because it may contribute to the pathogenesis of infertility or escalate a variety of infertility-related psychosocial problems. For instance, patients experiencing PCOS and infertility have a lower quality of life [11], and they are characterized by diminished marital satisfaction and sexual function [12].

Sexual function and sexual distress are interrelated symptoms that may stem from a certain degree of female sexual dysfunction (FSD). FSD, as a multidimensional symptomatology, is characterized by diminished levels of desire, genital arousal, orgasmic function, lubrication, and coital satisfaction, as well as painful intercourse [13]. Although transient and mild sexual difficulties are not sufficient to be considered sexual dysfunction disorders [14], recurrent or persistent difficulties that provoke sexual distress are deemed to be health issues [13, 15]. Sexual distress is described as negative emotional responses to sexual function impairment, such as anxiety, worry, discomfort, frustration, or feelings of inadequacy [16]. However, only up to half of patients with sexual dysfunction may exhibit sexual distress [15]. In addition, sexual distress may also occur without associated sexual dysfunction and may indicate reduced general well-being or depression symptomatology [28], which is prevalent in patients with comorbid PCOS [11, 17–19]. Thus, certain factors such as PCOS may contribute to the development of sexual distress in response to experiencing diminished sexual function in women with infertility.

To shed light on the association between sexual dysfunction and sexual distress in women with infertility, we suggest that the presence of PCOS may impose a greater association between sexual dysfunction and sexual distress. This suggestion is based on extant contradictory findings on the role of PCOS in female sexual disorders [20, 21]. Whereas some studies have shown the relationship between PCOS and sexual dysfunction [22], others have limited the effect of PCOS to certain domains of sexual function (e.g., desire and arousal) or even rejected any association [23, 24]. Most of these studies did not consider PCOS comorbid with infertility. In addition, it is important to account for the dimensionality of symptoms as a continuum (i.e., symptoms measured as a continuous variable), which best models an individual’s real-life experience. Thus, further research on the association between impaired sexual functioning and sexual distress is warranted, especially to determine whether PCOS is a risk factor for elevated sexual distress.

Overall, the role of sexual function in women’s personal, marital, and social lives is undeniable. Since sexual function plays an important role in quality of life, more attention should be given to the population of women with infertility and the comorbidity of PCOS. Therefore, the present study aimed to test the moderating role of PCOS in the relationship between sexual dysfunction and sexual distress in Iranian women with infertility.

## Materials and methods

### Design and setting

The current data were derived from a cross-sectional study conducted in 2018, which aimed to investigate biopsychosocial factors that contribute to sexual function of infertile patients with and without PCOS. Two groups of infertile female patients with and without PCOS who visited two infertility centers in Tehran, Iran, voluntarily participated in the study. Informed consent was obtained from all patients. To reduce the response burden, one resident and one nurse in the visiting centers used an interview-based method to collect data. The convenience sampling method was applied. A total of 190 women with infertility (PCOS = 103, non-PCOS = 87) completed the questionnaires.

The inclusion criteria were age over 18 years, a diagnosis of female infertility for all patients, and a PCOS diagnosis for only the PCOS group. Based on the international guidelines for polycystic ovary syndrome, patients with two or more conditions of androgen excess, ovulatory dysfunction, and ovarian morphology were identified as having PCOS [25]. When ovulatory dysfunction or androgen excess was not present, ultrasound was needed. Based on clinical judgment, specific conditions, including thyroid dysfunction (based on the level of thyroid-stimulating hormone (TSH)), late-onset congenital adrenal hyperplasia (based on the 17-hydroxyprogesterone level), and hyperprolactinemia (based on the prolactin level), were ruled out. Since these conditions can cause pseudo-PCOS symptoms, such as menstrual irregularities and androgen excess, by ruling out these conditions through specific hormone level tests, we can ensure that the symptoms observed were due to PCOS.

The exclusion criteria included pelvic endometriosis, external genitalia anomalies, current vaginal infection, pelvic mass, genitopelvic pain/penetration disorder, and chronic cardiovascular disease, which could otherwise affect sexual function. In addition, patients with serious emotional problems during the last six months (e.g., loss of primary family members, miscarriage) were excluded because recent negative life events could jeopardize sample homogeneity with respect to levels of depression and anxiety symptoms and related biological disturbance. Finally, the main study aimed to address the role of hormonal imbalance in the subsamples (e.g., testosterone), which required excluding patients who reported of the use of certain treatment-related medications, including gonadotropin-releasing hormone (GnRH) agonists, birth control pills for treatment purposes, or insulin sensitizers in the past six months. However, due to excessive missing data, we did not consider the role of hormones (i.e., covariate variable) in the current study.

### Applied measures

Demographics. The participants’ demographic information, consisting of the patient’s age, education and occupation, the husband’s age and education, and marital length, was collected through a checklist. Some relevant clinical information, including the duration of infertility and treatment, presence of abdominal obesity (assessed by waist-hip ratio), body mass index (BMI), and presence of hyperandrogenic symptoms, including acne, alopecia (i.e., androgenetic alopecia), and hirsutism (measured using the Ferriman-Gallway scoring system), was collected.

Sexual Function. The patients’ sexual function was assessed using the Female Sexual Function Index (FSFI) questionnaire [26]. This 19-item questionnaire assesses female sexual function across six domains, including desire (two items, domain factor 0.6), arousal (four items, domain factor 0.3), lubrication (four items, domain factor 0.3), orgasm (three items, domain factor 0.4), satisfaction (four items, domain factor 0.4), and pain (three items, domain factor 0.4), which comprehensively evaluate the female sexual response cycle. Each domain has a raw score, multiplied by its domain factor (forming a weighted score), resulting in a possible score ranging from 0 to 6, except for desire, where the range is between 1.2 and 6. The final score, which ranges from 1.2 to 36, is obtained by summing the weighted scores, with higher scores indicating better sexual function. The validity and reliability of the Farsi version of the FSFI have been previously established, with a test-retest reliability of 0.83 and a Cronbach’s alpha of 0.93 [27]. The instrument showed a Cronbach’s alpha of 0.90 in the current sample.

Sexual Distress. Female Sexual Distress Scale-Revised (FSDS-R), a 13-item self-report questionnaire, was used [28]. The Likert-type scale scores the items from NEVER (= 0) to ALWAYS (= 4). A higher total score indicates elevated sexual distress. The validity and reliability of the Farsi version of the FSDS-R have been previously established [29, 30]. The instrument showed a Cronbach’s alpha of 0.92 in the current sample.

Covariates. The Depression, Anxiety, and Stress Scale-21 (DASS-21) is a self-report tool containing three subscales—stress, depression, and anxiety—with seven questions apiece [31]. The items were scored based on a 4-point Likert-type scale ranging from NEVER (= 0) to VERY MUCH (= 3). The subscale score is the sum of the corresponding items. The validity and reliability of the Farsi version of the DASS-21 have been previously confirmed [32]. In the current study, only the DASS depression and anxiety subscales were included as covariate variables to account for the generalized contribution of current distress symptoms to sexual function and distress [33, 34].

### Data analysis

Descriptive statistics were reported for clinical and demographic variables using frequency, percentage, mean, and standard deviation and for study variables using mean and standard deviation for the total sample, PCOS subsample, and non-PCOS subsample. Pearson’s correlation coefficient was adopted to examine the paired relationships. Conditional process analysis was performed using the PROCESS macro v 4.5 for Statistical Package for Social Sciences (SPSS, IBM, Inc.) software [35]. Model 1 was utilized to test the moderation effect of PCOS as a dichotomous variable (yes versus no) on the relationship between sexual function as the independent variable and sexual distress as the dependent variable [36]. The models were examined as unadjusted and adjusted, with depression and anxiety as the covariate variables. We also investigated both the total FSFI (i.e., total sexual function) and the single domains covaried with the other domains to account for both the overall and the domain-specific associations.

The regression-based assumptions and diagnoses were examined where sexual distress was regressed on sexual functioning variables, depression and anxiety. The procedure indicated heteroscedasticity for lubrication (Koenker’s *P* = .015), satisfaction (Koenker’s *P* = .007), and orgasm (Koenker’s *P* = .037), for which heteroscedasticity-consistent standard errors (HC4) were applied. The analysis set for 5000 resamplings with 95% confidence intervals (CIs). Consistent with the current application of moderation analysis, a significant change in the slope of the main effect was inferred when the interaction term between the independent variable and moderator indicated *P* < .10 as the significance level. Data analysis was conducted using the Statistical Package for Social Sciences (SPSS, IBM, Inc.) software.

## Results

### Sample characteristics

Table 1 reports the sample’s clinical and demographic characteristics and group comparisons. On average, the demographic features of the subsamples were comparable, while those in the PCOS subsample were slightly younger (31.23 vs. 33.70 years, *P* = .003). The total sample was characterized by low education (diploma and lower, *n* = 105, 55.2%), increased BMI (26.56, 4.24), primary infertility (*n* = 88, 46.3), and predominantly housewife (*n* = 130, 68.4%).

**Table 1:** Clinical and demographic characteristics

Characteristics	Total sample (*N* = 190)			PCOS subsample (*n* = 103, 54.2%)			Non-PCOS subsample (*n* = 87, 48.7%)		Test Statistics	*p* value
**Patient’s age** (M, SD, range)	32.26 (5.39, 22–46)			31.23 (4.95, 23–46)			33.70 (5.66, 22–44)		t [163] = -2.97	0.003
**Spouse’s age** (M, SD, range)	36.19 (5.83, 26–52)			35.16 (5.29, 26–47)			37.59 (6.27, 27–52)		t [154] = -2.63	0.010
**Marital length** (M, SD, range)	6.66 (4.46, 1–22)			6.34 (4.23, 1–20)			7.09 (4.77, 1–22)		t [154] = -1.03	0.303
**Infertility length** (M, SD, range)	4.052 (3.39, 1–16)			3.789 (3.30, 1–14)			4.383 (3.49, 1–16)		t [147] = -1.07	0.289
**BMI** (M, SD)	26.56 (4.24)			26.56 (3.97)			26.56 (4.59)		t [159] = -0.002	0.998
**Central Obesity** (M, SD)	0.90 (0.06)			0.89 (0.06)			0.89 (0.06)		t [179] = 2.51	0.013
	n	%		n	%		n	%	*Chi*-squared	
**Infertility type**									2.42	0.298
Primary	88	46.3		53	51.5		35	40.2		
Secondary	56	29.5		27	26.2		29	33.3		
Not Specified	46	24.2		23	22.3		23	26.4		
**Education**									5.55	0.352
< Diploma	39	20.5		22	21.3		17	19.5		
Diploma	66	34.7		36	35.0		30	34.5		
University degree	49	25.8		31	30.1		18	20.7		
Not Specified	36	18.9		14	13.6		22	25.3		
**Job**									4.70	0.195
Housewife	130	68.4		72	69.9		58	66.7		
Work at House	2	1.1		2	1.9		0	0.0		
Employed	26	13.7		16	15.5		10	11.5		
Not Specified	32	16.8		13	12.6		19	21.8		
**Acne**									49.23	< 0.001
None	124	68.9		48	47.5		76	96.2		
Mild	42	23.3		39	38.6		3	3.8		
Moderate/severe	14	7.8		14	13.9		0	0		
**Baldness**									49.23	< 0.001
None	140	77.8		61	60.4		79	100.0		
Mild	23	12.8		23	22.8		0	0		
Moderate/severe	17	9.4		17	16.9		0	0		
**Hirsutism**									68.95	< 0.001
None	115	63.9		38	37.6		77	97.5		
Mild	42	23.3		40	39.6		2	2.5		
Moderate/severe	23	12.8		23	22.7		0	0		

Note. M: Mean. SD: Standard deviation. PCOS: Poly-cystic ovarian syndrome. BMI: Body mass index.

Table 2 also reports the descriptive statistics of the study variables and group comparisons. There were no significant group differences in the variables, although the PCOS group had elevated levels of depression (7.59 (7.49) vs. 5.84 (6.06), *P* = .08).

**Table 2:** Descriptive statistics of study variables

	Total Sample		PCOS sample		Non-PCOS sample		Skewness (S.E.=0.18)	Kurtosis (S.E.=0.351)	*P* value
Variables	M	SD	M	SD	M	SD			
Desire	3.78	1.07	3.77	1.05	3.79	1.10	− 0.15	− 0.71	0.906
Arousal	3.70	1.29	3.70	1.27	3.70	1.33	− 0.55	− 0.60	0.977
Lubrication	5.01	1.10	4.96	1.11	5.06	1.09	-1.27	1.32	0.513
Orgasm	4.62	1.07	4.54	1.18	4.71	0.93	− 0.73	− 0.03	0.270
Satisfaction	5.11	0.96	5.07	1.01	5.15	0.91	− 0.80	− 0.23	0.580
Pain	5.03	1.04	5.04	1.07	5.01	1.02	− 0.86	0.09	0.804
FSFI	27.24	4.40	27.09	4.88	27.42	3.77	− 0.47	− 0.39	0.607
Sexual distress	7.15	8.07	7.98	8.40	6.17	7.59	1.61	2.48	0.124
Depression	6.79	6.82	7.59	7.33	5.84	6.06	1.85	4.75	0.08
Anxiety	5.58	5.42	5.88	5.62	5.22	5.17	1.61	3.34	0.400

Note. M: Mean. SD: Standard deviation. PCOS: Poly-cystic ovarian syndrome. S.E.: Standard error. FSFI: Female sexual functioning inventory

### Paired correlations

Table 3 reports the Pearson’s correlation coefficient across the study variables. There were significant negative correlations between sexual function domains and sexual distress (*r* = − .27 ~ − .56, *P* < .001). Depression and anxiety were also correlated with sexual distress (*r* = .57, *P* < .001). The results indicated medium to large effect sizes.

**Table 3:** Pearson’s correlation coefficients between study variables (*N* = 190)

Variables	Desire	Arousal	Lubrication	Orgasm	Satisfaction	Pain	FSFI	Sexual distress
Sexual distress	− 0.37^***^	− 0.27^***^	− 0.43^***^	− 0.43^***^	− 0.50^***^	− 0.32^***^	− 0.56^***^	
Depression	− 0.24^**^	− 0.17^*^	− 0.31^***^	− 0.23^**^	− 0.30^***^	− 0.20^**^	− 0.35^***^	0.57^***^
Anxiety	− 0.17^*^	*− 0.12*	− 0.23^**^	*− 0.10*	− 0.20^**^	− 0.34^***^	− 0.28^**^	0.57^***^

Note. * *P* < .05, **P < .01, ***P < .001. FSFI: Female sexual functioning inventory

### Moderation analysis

**Fig. 1:**
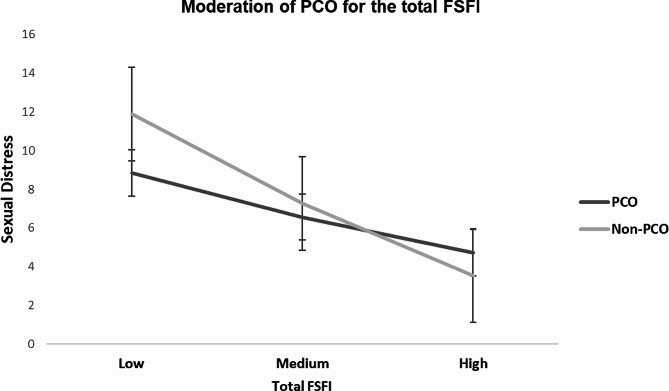
Moderating effect of PCOS on the adjusted relationship between the total FSFI score and sexual distress. Note that as the total FSFI score decreases, the problematic sexual function increases according to the FSFI scoring instructions

Table 4 reports the moderation analysis results for significant effects of adjusted models. According to both adjusted and unadjusted models for depression and anxiety symptoms, PCOS condition moderated the relationship of total FSFI with sexual distress such that decreased overall sexual function was associated with greater sexual distress in the PCOS group compared to their non-PCOS counterpart (*P* < .001, 95% CI=[-1.10, -0.63]) compared to the non-PCOS group (*P* = .008, 95% CI=[-0.74, -0.12]). (See Fig. 1).

**Table 4:** Moderation analysis of PCOS in the relationship between sexual function and sexual distress (*N* = 190)

IV	B	S.E.	*P* value	95%CI	
FSFI^a^	0.01	0.33	0.973	[-0.65, 0.67]	F = 45.01, *P* < .001, R^2^_adj_ = 0.54
PCOS	12.82	5.33	0.017	[2.32, 23.33]	
*Interaction*	-0.44	0.19	**0.023**	**[-0.82, -0.06]**	ΔR2 = 0.013, F = 5.24, *P* = .023
Pain^b^	2.97	1.25	0.018	[0.51, 5.43]	F = 25.04, *P* < .001, R^2^adj = 0.58
PCOS	11.07	3.96	0.006	[3.25, 18.89]	
*Interaction*	-2.07	0.77	**0.008**	**[-3.59, − 0.55]**	ΔR^2^ = 0.02, F = 7.19, *P* = .008

Note. IV: Independent variables. S.E: Standard error. CI = Confidence interval. FSFI: Female sexual functioning inventory. Bolded CIs indicate significant

^a^Including anxiety, depression as covariate variables

^b^Including anxiety and depression, and other sextual function domains as covariate variables

According to the domain-level analysis in the adjusted models for anxiety and depression, this difference could be attributed to desire (PCOS: *P* < .001, 95% CI=[-3.89, -1.64] vs. non-PCOS: *P* = .189, 95% CI=[-1.94, 0.39]), arousal (PCOS: *P* < .001, 95% CI=[-2.94, -1.03] vs. non-PCOS: *P* = .699, 95% CI=[-1.16, 0.78]), and pain (PCOS: *P* < .001, 95% CI=[-3.53, -1.18] vs. non-PCOS: *P* = .569, 95% CI=[-0.91, 1.65]).

**Fig. 2:**
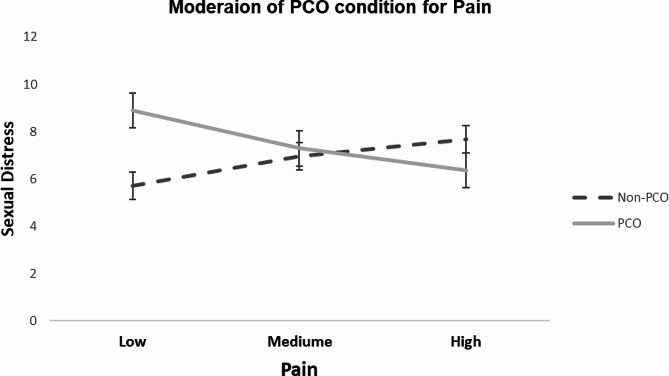
Moderating effect of PCOS on the adjusted relationship between sexual pain and sexual distress. Note that as the pain score decreases, the problematic pain function increases according to the FSFI scoring instructions

When the domain-level analysis was further adjusted for the remaining domains, only the effect of decreased sexual pain function on sexual distress was moderated by PCOS condition (*P* = .008, 95% CI=[-3.59, − 0.55]), with a significant downward effect for PCOS (B = -1.16, *P* = .04, 95% CI=[-2.26, -0.05] compared to a non-significant upward effect for non-PCOS group (B = 0.91, *P* = .122, 95% CI=[-0.24, 2.06]) (see Fig. 2).

## Discussion

The present study tested whether PCOS could moderate the association between diminished sexual function and increased sexual distress in women with infertility. The results suggested that women with PCOS may have greater sexual distress when their sexual functioning is impaired. Specifically, where PCOS exists, there is greater sexual distress due to diminished sexual pain function and, to some extent, decreased arousal and desire function. Importantly, controlling for depression and anxiety symptoms, the current study highlights the role of PCOS in women with infertility, in which decreased sexual pain function is associated to elevated sexual distress.

A recent meta-analysis suggested that PCOS does not directly increase the risk of FSD [21]. Likewise, our results indicate that although the rate of diminished sexual function in infertile patients with PCOS is similar to that in infertile patients without PCOS, infertile patients with PCOS experience relatively greater sexual distress due to impaired sexual desire, arousal, and pain. Specifically, the effect of depression and anxiety symptoms on sexual function accounted for [37, 38], a higher reduction in sexual pain function was associated with elevated sexual distress in PCOS patients, but not in their non-PCOS counterpart. In other words, depression and anxiety symptoms may contribute to elevated sexual distress due to impaired sexual desire and arousal in infertile patients regardless of their PCOS status. However, PCOS, not depression or anxiety, makes infertile patients prone to elevated sexual distress due to increased sexual pain.

Despite the biological, context dependent, and psychosexual etiology of pain in heterosexual penile-vaginal intercourse, PCOS patients experience subjective concern about having genito-pelvic pain/penetration disorder [39]. Sexual pain is the most impaired sexual function in Iranian women with infertility [7]. However in the population with PCOS, the literature reports inconsistent findings from significantly lower [40] to intact values [41]. Considering that PCOS is comorbid with infertility, the current study suggested that increased sexual pain is linked to elevated sexual distress. Notably, sexual pain is deemed to be a multidimensional phenomenon with both psychosocial and biological roots [42]. Thus, one may suggest that PCOS may contribute to a set of intervening physical and psychological factors that underlie the link between sexual pain and sexual distress [43]. Further research may elucidate the biopsychosocial factors involved in this phenomenon.

Supported by religious views, childbearing is highly honored, and Muslims believe that “heaven lies at the feet of *mothers*” [44]. Consequently, infertility imposes great pressure on couples and increases their susceptibility to psychological distress [45]. A qualitative study in Iran demonstrated that women with infertility experience social pressure, directed chiefly by close relatives and in-laws, for their infertility [46]. The current study indicated that diminished sexual function is associated with sexual distress in Iranian women with infertility, and infertile women patients with comorbid PCOS may suffer from relatively higher associated sexual distress. Therefore, we call for special attention to address these patients’ psychosocial needs, rather than solely relying on medication-based treatment plans (e.g., inositol and metformin) [47, 48]. In fact, the management of sexual function in infertility comorbid with PCOS requires multidimensional approach in which the associated complications, such as obesity and insulin resistance are also included [48, 49].

The study findings revealed the importance of considering sexual distress in the treatment management for infertile patients with PCOS. The extant literature on the role of PCOS in developing sexual dysfunction indicates comparable levels of sexual dysfunction (i.e., the FSFI total score) compared with healthy controls [21]. Our findings suggest that PCOS condition moderates the dynamic among sexual function challenges and the patients’ psychological responses (i.e., distress), such that PCOS condition exacerbates the associated sexual distress in infertile women patients. Accordingly, the therapeutic team, including nurses, psychotherapists, gynecologists, and sex therapists addressing sexual dysfunction, especially sexual pain, may need to pay special attention to sexual distress as a psychological consequence of PCOS. It is recommended that future studies address the underlying trajectories of pain in women with infertility and PCOS comorbidity, in which they may consider the effect of psychological factors (e.g., an individual’s response to anticipation of pain during sex) as well as physical determinants [43].

The empirical results reported herein should be considered in light of several limitations. A general limitation is that due to the cross-sectional nature of the study, caution should be taken before generalization and causal implications can be achieved. Further prospective research is required to examine the link between FSD and sexual distress in terms of PCOS. Our study findings might also be affected by the sample size, which lowered the study power. Thus, the findings should be interpreted with caution. In addition, the current findings might be biased by positive sexual attitudes and sexual experience, which have been shown to be predictive factors for willingness to volunteer in sexual studies [50]. This study lacked a control group of women without infertility, so it failed to examine any possible differences from the general population. Lacking robust data regarding patients’ hormonal profile, likewise, we could not account for the effect of hormone levels on sexual dysfunction with respect to PCOS comorbidity. However, we minimized the effect of confounding factors by excluding conditions that could independently affect sexual function. In addition, the study population (Iranian patients) offers culturally diverse insights for the literature. Furthermore, our moderation analysis deepened our understanding of the interplay between PCOS, sexual dysfunction, and sexual distress.

Hyperandrogenaemia may have a negative effect on sexual function [51]. Conversely, however, some studies have indicated that hyperandrogenism could act as a protective agent for sexual functioning by enhancing sexual desire and the frequency of satisfying sexual activity [52, 53]. Generally, this hypothesis is worthy of further research because hormonal imbalance may impact sexual distress through factors such as alopecia, hirsutism, and subsequent poor body image resulting from high testosterone levels in PCOS patients [54]. Furthermore, considering that the current PCOS sample was slightly younger than their non-PCOS counterparts, some studies have shown the mitigating effect of older age on the association between sexual dysfunction and sexual distress in the general population [34]. Due to the multicausal nature of sexual dysfunction and sexual distress, a more coordinated assessment of psychosocial, hormonal and biological aspects is necessary. Finally, the relationship between sexual dysfunction and sexual distress may be reciprocal because not only can sexual dysfunction lead to sexual distress, but sexual distress might also incite sexual dysfunction [15]. The current study could not address this suggestion.

## Conclusions

The current study showed that infertility comorbid with PCOS renders women susceptible to sexual distress where sexual pain is impaired. Hence, it is suggested that gynecologists consider the psychological experience of patients, and researchers should extend these investigations to identify the network of psychological factors in addition to the physiological agents that affect sexual distress. The contribution of PCOS to distressing sexual pain warrants further research.

## Data Availability

The datasets used and/or analyzed during the current study are available from the corresponding author upon reasonable request.
